# Efficacy and safety of sonothombolysis versus non-sonothombolysis in patients with acute ischemic stroke: A meta-analysis of randomized controlled trials

**DOI:** 10.1371/journal.pone.0210516

**Published:** 2019-01-09

**Authors:** Zhouqing Chen, Tao Xue, Huachen Huang, Jiayi Xu, Sandhya Shankar, Hao Yu, Zhong Wang

**Affiliations:** 1 Department of Neurosurgery & Brain and Nerve Research Laboratory, The First Affiliated Hospital of Soochow University, Suzhou, Jiangsu Province, China; 2 Department of Neurology, First affiliate Hospital, Harbin Medical University, Harbin, Heilongjiang Province, China; 3 Department of Ophthalmology, The First Affiliated Hospital of Soochow University, Suzhou, Jiangsu Province, China; 4 Department of Neurology, University of Pittsburgh, Pittsburgh, Pennsylvania, United States of America; 5 Department of Neurosurgery, The First People’s Hospital of Nantong city, Nantong, Jiangsu Province, China; Universita degli Studi di Roma La Sapienza, ITALY

## Abstract

Recent studies have shown that inconsistent results of safety and efficacy between sonothombolysis vs. non-sonothombolysis in acute ischemic stroke (AIS). We implemented a meta-analysis to explore the value of sonothrombolysis in AIS treatment. The MEDLINE, EMBASE, and Cochrane Library databases were searched for randomized controlled trials (RCTs) which had evaluated sonothrombolysis or ultrasound thrombolysis in AIS. One hundred five studies were retrieved and analyzed, among them, 7 RCTs were included in the current meta-analysis. In comparison with the non-sonothombolysis, sonothrombolysis significantly improved complete recanalization (RR 2.16, 95% CI 1.51 to 3.08, *P* < 0.001), complete or partial recanalization (RR 1.90, 95% CI 1.26 to 2.88, *P* = 0.002), there is also a tendency to improvement of ≥ 4 points in NIHSS score (RR 1.43, 95% CI 0.99 to 2.07, *P* = 0.057). However, sonothrombolysis and non-sonothrombolysis had insignificant differences in neurological recovery and adverse events. In subgroup analysis, we found that “With t-PA”, “NIHSS > 15”, “Treatment time ≤ 150min”, and “Age ≤ 65 years” are potential favorable factors for efficacy outcomes of sonothombolysis. Sonothrombolysis can significantly increase the rate of recanalization in patients with AIS compared with non-sonothrombolysis, but there is no significant effect on improving neurological functional recovery and avoiding complications.

## Introduction

Stroke, along with cardiac disease and cancer are the most common reasons for permanent disability, which is the second leading cause of death worldwide among them [1, 2]. Ischemic stroke is characterized by the partial or complete loss of blood supply in part of the brain tissues and then causes dysfunction which occupies approximately 70–80% of all kinds of strokes [2, 3]. So far, when a sudden arterial occlusion occurs, followed by cerebral ischemic stroke, mechanical thrombolysis, and intravenous thrombolysis are the dominating methods to get recanalization, reduce the severity, and improve the outcomes [4]. In addition, sonothrombolysis is a novel method for the treatment of acute ischemic stroke (AIS). Sonothrombolysis by transcranial Doppler (TCD) or transcranial color-coded sonography (TCCS) with or without t-PA is a potential, promising, but debatable, the method for treating AIS patients [5]. Ultrasound can accelerate the blood flow near the thrombus, so we assumed that the application of TCD/TCCS could stir up the blood near the obstructed thrombus [6]. This way can also enhance the mixture of t-PA into a blood and increase the concentration of the t-PA near the occlusion consequently [7]. In addition, the pressure waves produced by TCD/TCCS may also augment the permeation of t-PA into the fibrin network and affect the binding of t-PA with fibrin directly. [8]

Microbubbles are air- or gas-filled microspheres [9] which were initially used to improve the echo-graphic quality of images by increasing the acoustic signal [10]. Application of TCD/TCCS has been shown to cause the vibration of microbubbles, resulting in the microbubbles sustaining absorption of energy until they explode and release the energy [11]. Hence, the ultrasound-induced blast of microbubbles may speed up the dissolution of thrombus. Microbubbles combined with TCD/TCCS were also applied to some other clinical trials, which might be able to get better clinical outcomes [12].

Recent years, there are several trials have consistently illustrated that sonthrombolysis can improve the outcomes of AIS [9, 13–17]. Eggers, J., et al. 2005 [14], Eggers, J., et al. 2008 [15] and Dwedar, A.Z., et al. 2014 [16] demonstrated that sonothrombolysis has positive effects on increasing the recanalization rate and improve the clinical outcomes of acute middle cerebral artery occlusion. CLOTBUST 2004 [17] and Molina, C.A., et al. 2006 [9] found that continuous TCD improves t-PA-induced arterial recanalization in patients with AIS, which did not significantly help patients to recover. TUCSON 2009 [13] concluded that ultrasound combined with microbubbles and t-PA showed higher recanalization rates and more favorable outcome rates compared with intravenous t-PA therapy alone. NOR-SASS 2017 [18] revealed that sonothrombolysis is safe, but there is no statistically significant clinical effect in unselected AIS patients.

Based on the above-mentioned clinical studies and trials, the efficacy and safety of sonothrombolysis for AIS are still unclear. There were been some similar systematic reviews that discuss sonothrombolysis in the treatment of AIS [5, 19]. However, our work represents an updated of the previous meta-analysis according to the publication of two novel studies in 2014 [16] and 2017 [18]. In addition, the current meta-analysis explores the impact of relevant factors on sonothrombolysis by more comprehensive subgroup analysis.

## Materials and methods

### Study protocol

This meta-analysis was written according to the preferred reporting items for systematic reviews and PRISMA statement (S1 File) [20]. At the start of this project, a drafted study protocol was made to consist with the Cochrane Collaboration format [21].

### Eligibility criteria

Inclusion criteria were as follows: (a) Study type: RCT; (b) Language restriction: only English was available; (c) Participants: patients with AIS; (d) Intervention: sonothrombolysis; (e) Outcomes: Efficacy outcomes: excellent functional outcome (modified Rankin Scale (mRS) = 0–1) and good functional outcome (mRS = 0–2) based on mRS, early neurological improvement based on NHISS (NIHSS improve ≥ 4) and recanalization; Safety outcomes: intracranial hemorrhage, death (mRS = 6) and disability (mRS = 3–5). (f) Study years: We searched MEDLINE, EMBASE, and Cochrane Library to find related articles from January 2001 to May 2018. Exclusion criteria were as follows: (a) Study types: case reports, case reviews, retrospective studies, and cohort studies; (b) Control: positive control; (c) Conference abstracts without full text.

### Search strategy and information sources

Three major databases: MEDLINE, EMBASE, and Cochrane Library were systematically searched by two authors independently (Z.C. and T.X.). The search strategy of the MEDLINE was to combine all the variables [(“ultrasound” AND “thrombolysis”) OR “sonothrombolysis”] AND “acute ischemic stroke.” Two independent investigators (Z.C. and T.X.) scanned the titles and abstract of all the studies to select applicable studies. The search strategy for EMBASE and the Cochrane Library is similar to what we used for searching MEDLINE. In addition, two investigators (Z.C. and T.X.) manually screened reference lists from RCTs and systematic reviews independently to ensure all relevant studies have been included in this study.

### Study selection and data collection

All records from the systematic search in the electronic database and reference lists of RCTs and systematic reviews were evaluated by two authors (Z.C. and T.X.) independently following the eligibility criteria as mentioned above. After strict selection and evaluation, we collect the data from RCTs as follows: basic information on the included trials, inclusion, exclusion criteria for the participants, study design, and outcome assessments (Table 1).

**Table 1:** Baseline characteristics of the included studies and outcome events in the meta-analysis.

Trials	Therapeutic centre	Publication	Inclusion Criteria	Exclusion Criteria	Study Design	Efficacy outcomes	Safety outcomes
**Alexandrov 2004**	5 centers in 3 countries	***N Engl J Med***	NIHSS score ≥ 4; No evidence of hemorrhage; Intravenous tPA infusion initiated within 3 hours of symptom onset; Diagnostic TCD completed before TPA bolus showing arterial occlusion.	Absent temporal windows; Primary intra-arterial thrombolysis; Patient refusal to give informed consent to participate in the CLOTBUST trial; Standard contraindications for intravenous tPA therapy.	2MHz TCD + tPA vs. tPAc	CR in 2 hours Clinical recovery in 2 hours NIHSS scores’ improvement mRS scores at 3 months	Reocclusion in 2 hours Death and disability at 3 months
**Eggers 2005**	2 centers in 1 country	***Neurology***	Ischemic stroke in the MCA territory as diagnosed within 6 hours of symptom onset; MCA-M1 occlusion diagnosed by TCCS; Contraindications for IV thrombolytic therapy according to protocol of NINDS rt-PA Stroke Study.	Thrombolytic therapy if they show EICs greater than one third of the MCA territory; Subjects who met the criteria of the protocol of the NINDS and had EICs in less than one third of the MCA territory.	TCCS vs. none	CR, PR, NR in 1 hour and 24 hours NIHSS scores’ improvement at 24 hours and 4 days mRS scores at 3 months	Death at 3 months and follow-up ICH
**Molina 2006**	6 centers in 3 countries	***Stroke***	Patients with acute ischemic stroke admitted within the first 3 hours after symptom onset; Patients with a nonlacunar stroke involving the vascular territory of the MCA; Patients underwent urgent carotid US and TCD examinations.	Insufficient acoustic temporal window or absence of any residual flow in the MCA for TCD examination; Taking anticoagulants, experienced dramatic spontaneous neurological improvement, or showed early signs of infarction< 33% of the MCA territory on baseline CT.	2MHz TCD + tPA +MB vs. 2MHz TCD + tPA vs. tPA alone	CR, PR, NR in 2 hours NIHSS scores’ improvement at 24 hours mRS scores at 3 months	Disability at 3 months ICH
**Eggers 2008**	4 centers in 1 country	***Stroke***	Ischemic stroke in MCA territory within 3 hours of symptom onset; Early ischemic changes one third or less of the MCA territory; Thrombolytic therapy in accordance with the NINDS; MCA-M1 occlusion diagnosed by TCCS;	Age <18 years or >80 years; 2.Pregnancy or lactation; Premorbid mRS >1; Insufficient acoustic window.	1.8 MHz TCCS + tPA vs. tPA	CR, PR, NR in 1 hour and 24 hours NIHSS scores’ improvement at 24 hours and 4 days BI>95 at 3 months mRS scores at 3 months	Death and disability at 3 months and follow-up ICH
**Molina 2009**	10 centers in 6 countries	***Annals of Neurology***	All acute (<3 hours) ischemic stroke patients who had a disabling neurological; A proximal intracranial arterial occlusion documented by a TIBI flow score of 0–3 in either the MCA, ACA, ICA, PCA, or top-of-the-distal basilar artery on baseline TCD assessment.	Evidence of hemorrhage on noncontrast head; Contraindication for intravenous tPA therapy; Primary treatment with intra-arterial thrombolysis; Absent temporal insonation windows; 5. Related contraindications	2MHz TCD + tPA + uS vs. tPA	CR, PR, NR in 2 hours Clinical recovery in 2 hours mRS scores at 3 months	Reocclusion in 2 hours Death and disability at 3 months ICH
**Dwedar 2014**	1 center in 1 country	***Neurology India***	Patients with acute ischemic stroke in the MCA territory within 24 h of onset of symptoms; Age ranging between 40 and 70 years.	Onset of stroke more than 24h; Poor acoustic window; Patients intolerant to monitoring.	2MHz TCD + aspirin vs. aspirin	CR, PR, NR in 1 hour NIHSS scores’ improvement at 24 hours and 7 days MFV changes	
**Nacu 2017**	5 centers in 1 country	***Stroke***	Patients with acute ischaemic stroke, with or without a visible arterial occlusion; Start of treatment within 4.5 hours after stroke onset; Age >18 years.	Premorbid mRS score ≥ 3; NIHSS cannot be obtained; No visible occlusion; Intracranial haemorrhage; Clinical presentation suggesting subarachnoid haemorrhage; Large areas of hypodense ischaemic changes; Any other serious medical illness likely to interact with treatment.	2Mhz TCD + tPA +MB vs. tPA	CR and NR in 24 hours NIHSS scores’ improvement at 24 hours mRS scores at 3 months	Death and disability at 3 months and follow-up ICH

**CLOTBUST**: Combined Lysis Of Thrombus in Brain ischemia Using transcranial ultrasound and Systemic TPA; **TPA**: tissue plasminogen activator; **TCD**: transcranial Doppler; **TIBI**: Thrombolysis In Brain Ischemia; **NIHSS**: National Institute of Health Stroke Scale; **mRS**: modified Rankin scores; **CR**: complete recanalization; **PR**: partial recanalization; **NR**: no recanalization; **NINDS**: National Institute of Neurologic Disorders and Stroke; **EICs**: early ischemic changes; **TCCS**: transcranial color-coded sonography; **MB**: Microbubble; **ICH**: intracerebral hemorrhage; **MCA**: the middle cerebral artery; **ACA**: anterior cerebral artery; **ICA**: internal carotid artery; **PCA**: posterior cerebral artery; **BI**: barthel index; **TUCSON**: Transcranial Ultrasound in Clinical SONothrombolysis; **uS**: Microspheres; **NOR-SASS**: NORwegian Sonothrombolysis in Acute Stroke Study; **MFV**: mean flow velocity.

### Risk of bias

The risk of bias plot in individual studies was created using the Review Manager 5.2 software. Uniform criteria of the Cochrane collaboration we applied to assess the risk of bias of RCTs, including selection bias, performance bias, detection bias, attrition bias, reporting bias, and some other potential biases.

### Summary measures and synthesis of results

STATA (Version 12.0) was used for assessing the data. Dichotomous outcomes were analyzed as the risk ratio (relative risk [RR]; 95% confidence interval [CI]) and calculated using a random effect model. Statistical heterogeneity was estimated by the *I*^2^ statistic as follows: *I*^2^ < 30% means “low heterogeneity” *I*^2^ = 30 to 50% denotes “moderate heterogeneity,” and *I*^2^ > 50% represents “substantial heterogeneity.” Subgroup analyses were implemented to detect the application of microbubble and t-PA, the severity of Stroke, time from onset to treatment and patients’ ages. Sensitivity analysis was used to explore the stability of the consolidated results. Two-tailed test and a P value less than 0.05 was considered significant for all analyses.

## Results

**Fig 1:**
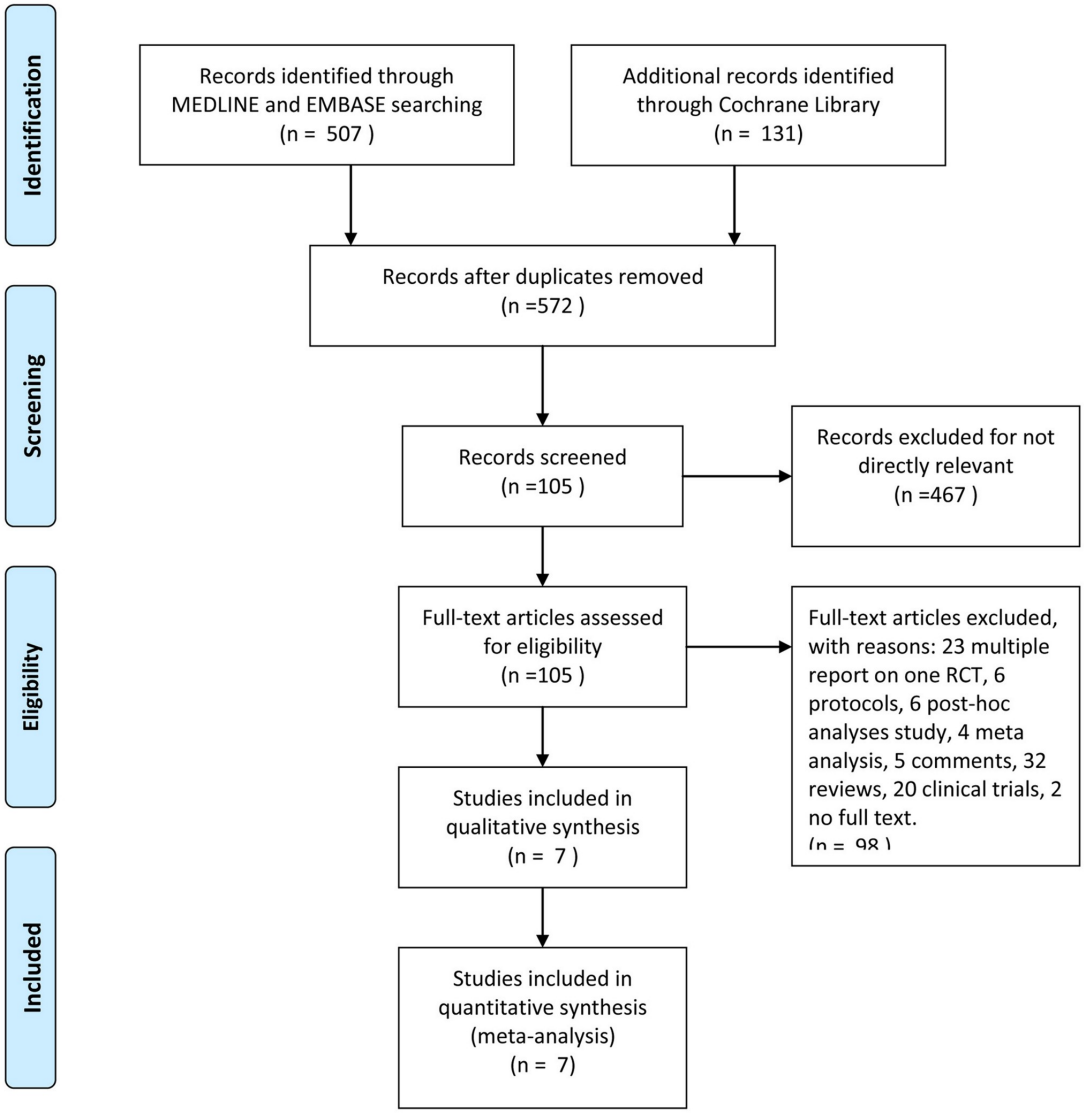
The study search, selection, and inclusion process.

Six hundred thirty-eight titles and abstracts were identified through MEDLINE, EMBASE, and Cochrane Library in total (Fig 1). After removing the duplicates and irrelevant records, 105 articles were included, and 98 articles were excluded because of their types: 23 multiple reports on one RCT, 6 protocol studies, 6 post-hoc analysis, 4 meta-analysis, 5 comments, 32 reviews, and 20 nonrandomized clinical trials. Additionally, 2 conference abstracts without full-text Larrue, V., et al. 2007 [22] and Dinia, L., et al. 2016 [23] were also excluded from the current meta-analysis. Furthermore, the trial of Alexandrov et al. 2008 [24] was excluded due to lose the follow-up information of some patients at three-month (9 out of 15, or 60%) and the data of non-ultrasound control group was the same as CLOTBUST 2004 [17]. Ultimately, seven RCTs [9, 13–18] were eligible and contained 549 patients were included in qualitative synthesis (Fig 1). The main characteristics of those included studies are listed in Table 1.

### Outcomes analysis

All 7 RCTs [9, 13–18] include 549 patients are available for the analysis of efficacy and safety outcome.

#### 1. Efficacy outcome

**Fig 2:**
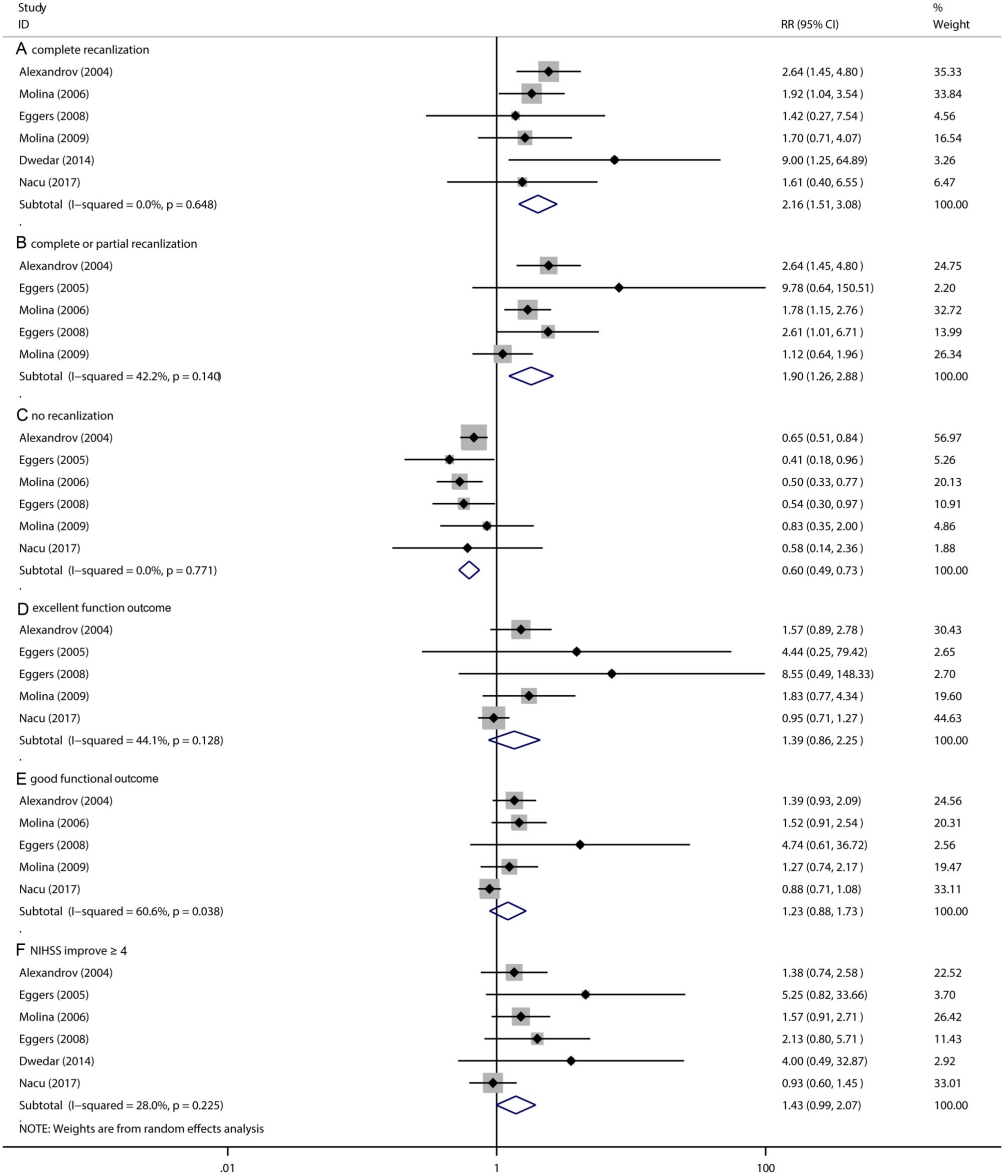
The pooled relative risk of the efficacy outcomes. The diamond indicates the estimated relative risk (95% confidence interval) for all patients together. A, Complete recanlization. B, Complete or partial recanlization. C, No recanlization. D, Excellent function outcome. E, Good functional outcome. F, NIHSS improve ≥ 4.

Sonothrombolysis group has an advantage over the non-sonothrombolysis group for patients in increasing the complete recanalization (RR 2.16, 95% CI 1.51 to 3.08, *P* < 0.001; Fig 2A), complete or partial recanalization (RR 1.90, 95% CI 1.26 to 2.88, *P* = 0.002; Fig 2B) and decreasing the no recanalization (RR 0.60, 95% CI 0.49 to 0.73, *P* < 0.001; Fig 2C). In addition, these was no significant difference between the excellent functional outcome (RR 1.39, 95% CI 0.86 to 2.25, *P* = 0.181; Fig 2D) and the good functional outcome (RR 1.23, 95% CI 0.88 to 1.73, *P* = 0.229; Fig 2E), which respectively were defined as the 3-month mRS = 0–1 or 0–2. However, compared with non-sonothrombolysis group, sonothrombolysis group also have a tendency, which can decrease NIHSS score more than 4 in AIS patients (RR 1.43, 95% CI 0.99 to 2.07, *P* = 0.057; Fig 2F). The heterogeneity test showed significant differences among studies (*I*^2^ = 60.6%, *P* = 0.038). (Fig 2E). To detect the source of the statistical heterogeneity, sensitivity analysis was performed. The sensitivity analysis showed that all of the consolidated results were stable (S1 Fig).

#### 2. Safety outcome

**Fig 3:**
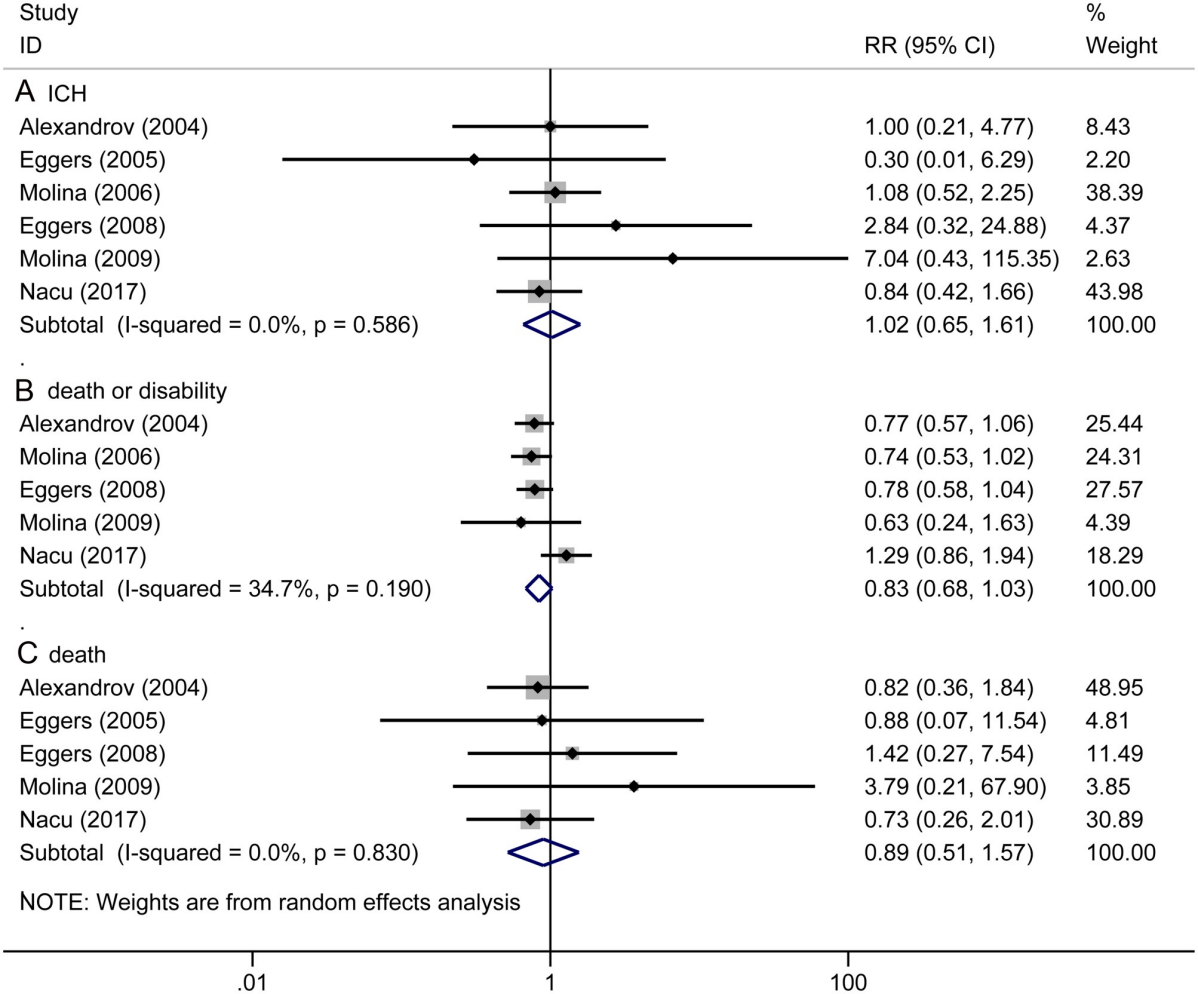
The pooled relative risk of the safety outcomes. The diamond indicates the estimated relative risk (95% confidence interval) for all patients together. A, ICH. B, Death or disability. C, Death.

There exist no significant differences in preventing the adverse events, including: ICH (RR 1.02, 95% CI 0.65 to 1.61, *P* = 0.923, Fig 3A), death or disability (RR 0.83, 95% CI 0.68 to 1.03, *P* = 0.091, Fig 3B), death (RR 0.89, 95% CI 0.51 to 1.57, *P* = 0.698, Fig 3C) between sonothrombolysis group and control group.

### Subgroup analysis

We performed subgroup analyses to assess the utility of microbubbles or t-PA combined with TCD/TCCS, the severity of stroke at the beginning, time of initial treatment and age of patients.

#### 1. With/without microbubble

The subgroup without microbubble in sonothrombolysis group had a higher rate of excellent functional recovery (RR 1.74, 95% CI 1.00 to 3.01, *P* = 0.049; Table 2), good functional outcome (RR 1.43, 95% CI 1.03 to 1.99, *P* = 0.032; Table 2), NIHSS improvement ≥ 4 (RR 1.60, 95% CI 1.09 to 2.36, *P* = 0.017; Table 2) and simultaneously reduced the statistics of death or disability (RR 0.79, 95% CI 0.65 to 0.95, *P* = 0.011; Table 2) compared with non-sonothrombolysis group.

**Table 2:** Subgroup analysis of efficacy and safety outcomes.

	**Efficacy outcomes**					
	Excellent functional outcome		Good functional outcome		NIHSS improve ≥ 4	
	RR (95% CI)	P value	RR (95% CI)	P value	RR (95% CI)	P value
**1.use microbubble**						
Yes	1.155 (0.637, 2.095)	0.635	1.154 (0.762, 1.747)	0.499	1.267 (0.663, 2.419)	0.474
No	1.738 (1.002, 3.014)	0.049	1.432 (1.031, 1.988)	0.032	1.600 (1.086, 2.356)	0.017
**2.use tPA**						
Yes	1.349 (0.825, 2.207)	0.233	1.231 (0.877, 1.728)	0.229	1.284 (0.930, 1.771)	0.129
No	4.444 (0.249, 79.42)	0.311	N/A	N/A	4.661 (1.157, 18.78)	0.030
**3. Stroke severity at baseline**						
NIHSS ≤ 15	1.215 (0.681, 2.167)	0.511	0.967 (0.701, 1.333)	0.838	2.013 (0.556, 7.288)	0.287
NIHSS > 15	2.081 (0.584, 7.420)	0.259	1.481 (1.081, 2.028)	0.015	1.568 (1.074, 2.290)	0.020
**4.time to treatment (mins)**						
Time ≤ 150	1.720 (1.075, 2.754)	0.024	1.387 (1.007, 1.909)	0.045	1.566 (0.925, 2.650)	0.095
Time > 150	1.044 (0.494, 2.208)	0.909	1.100 (0.631, 1.920)	0.736	1.480 (0.814, 2.690)	0.199
**5.Age (years)**						
Age ≤ 65	6.185 (0.814, 47.01)	0.078	4.737 (0.611, 36.72)	0.137	2.767 (1.237, 6.187)	0.013
Age > 65	1.247 (0.809, 1.923)	0.318	1.178 (0.859, 1.616)	0.308	1.211 (0.869, 1.686)	0.258
	**Safety outcomes**					
	ICH		Death or disability		Death	
	RR (95% CI)	P value	RR (95% CI)	P value	RR (95% CI)	P value
**1.use microbubble**						
Yes	1.064 (0.587, 1.929)	0.838	0.866 (0.517, 1.449)	0.583	0.950 (0.282, 3.197)	0.934
No	1.027 (0.512, 2.060)	0.940	0.786 (0.653, 0.946)	0.011	0.906 (0.450, 1.826)	0.783
**2.use tPA**						
Yes	1.052 (0.665, 1.663)	0.830	0.835 (0.678, 1.029)	0.091	0.895 (0.501, 1.598)	0.708
No	0.296 (0.014, 6.292)	0.435	N/A	N/A	0.875 (0.066, 11.54)	0.919
**3. Stroke severity at baseline**						
NIHSS ≤ 15	1.025 (0.293, 3.585)	0.969	1.030 (0.534, 1.986)	0.930	0.872 (0.355, 2.144)	0.765
NIHSS > 15	1.158 (0.615, 2.183)	0.650	0.766 (0.641, 0.916)	0.003	0.909 (0.439, 1.882)	0.797
**4.time to treatment (mins)**						
Time ≤ 150	1.875 (0.591, 5.947)	0.286	0.770 (0.625, 0.948)	0.014	0.990 (0.489, 2.005)	0.977
Time > 150	0.916 (0.559, 1.499)	0.726	0.965 (0.548, 1.698)	0.901	0.744 (0.289, 1.919)	0.541
**5.Age (years)**						
Age ≤ 65	1.198 (0.139, 10.32)	0.870	0.780 (0.583, 1.044)	0.095	1.232 (0.303, 5.001)	0.771
Age > 65	1.004 (0.628, 1.605)	0.987	0.859 (0.644, 1.145)	0.299	0.840 (0.453, 1.559)	0.580

**RR**: Relative Risk; **CI**: Confidence Interval; **N/A**: Not Applicable.

#### 2. With/without t-PA

The subgroup without t-PA in sonothrombolysis group had a more effective NIHSS improvement (RR 4.66, 95% CI 1.16 to 18.78, *P* = 0.030; Table 2) compared with non-sonothrombolysis group. However, the subgroup with t-PA in sonothrombolysis group might be safer compared with non-sonothrombolysis group by showing a trend towards the attenuation of death or disability (RR 0.84, 95% CI 0.68 to 1.03, *P* = 0.091; Table 2).

#### 3. NIHSS > 15 / NIHSS ≤ 15 at the beginning of stroke

The high stroke severity subgroup, patients’ NIHSS > 15 at the beginning of stroke in sonothrombolysis group was more likely to get good functional outcome (RR 1.48, 95% CI 1.08 to 2.03, *P* = 0.015; Table 2) and NIHSS improvement ≥ 4 (RR 1.57, 95% CI 1.07 to 2.29, *P* = 0.020; Table 2), also had less death or disability (RR 0.77, 95% CI 0.64 to 0.92, *P* = 0.003; Table 2) compared with non-sonothrombolysis group.

#### 4. Time of the initial treatment > 150min / ≤ 150min

The early treatment subgroup (≤ 150min) in sonothrombolysis group exhibited significantly differences in elevating excellent functional outcome (RR 1.72, 95% CI 1.08 to 2.75, *P* = 0.024; Table 2), good functional outcome (RR 1.39, 95% CI 1.01 to 1.91, *P* = 0.045; Table 2) and lowering death or disability (RR 0.77, 95% CI 0.63 to 0.95, *P* = 0.014; Table 2), compared with non- sonothrombolysis group.

#### 5. Age > 65 years / ≤ 65 years

The patients ≤ 65 years subgroup in sonothrombolysis group were easier to get NIHSS improvement ≥ 4 (RR 2.77, 95% CI 1.24 to 6.19, *P* = 0.013; Table 2) and a tendency to get more excellent functional outcome (RR 6.19, 95% CI 0.81 to 47, *P* = 0.078; Table 2) and less death or disability (RR 0.78, 95% CI 0.58 to 1.04, *P* = 0.095; Table 2) compared with non- sonothrombolysis group.

The sensitivity analysis illustrated that all the consolidated statistics are stabilized.

### Risk of bias in included studies

**Fig 4:**
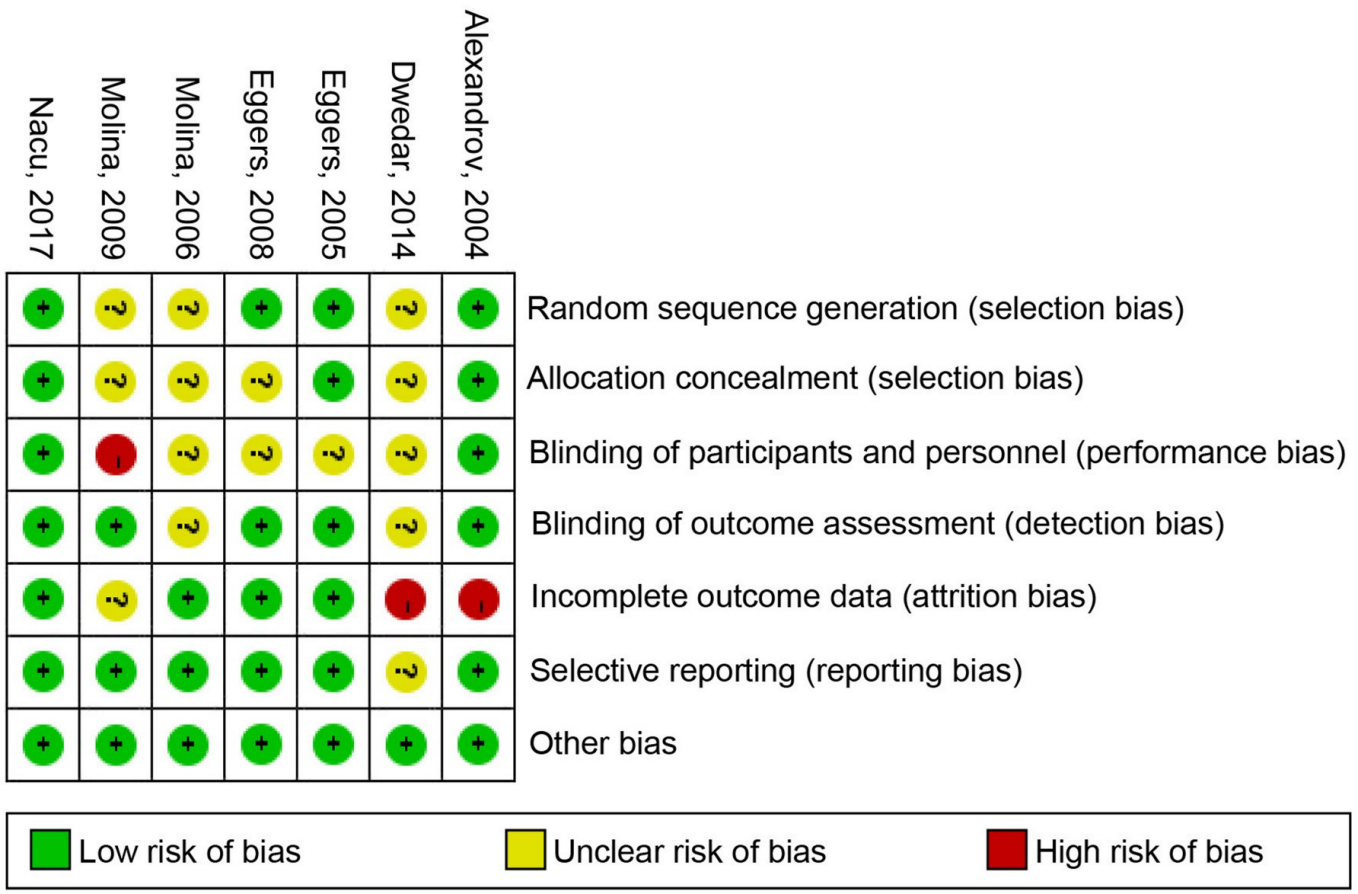
**Risk of bias**: A summary table for each risk of bias item for each study.

Details about the risk of bias of the included studies are shown in Fig 4. For the random sequence generation assessment, the risk of bias was unclear in 3 out of 7 studies. For the allocation concealment assessment, the risk of bias was unclear in 5 out of 7 studies. For the blinding of participants and personnel assessment, the risk of bias of four trial was unclear and 1 out of 7 trial had a high risk of bias. For the blinding of outcomes assessment, the risk of bias was unclear in 2 out of 7 studies. For incomplete outcome data, the risk of bias of 1 out of 7 trial was unclear and 2 out of 7 trials had a high risk of bias. For the selective reporting assessment, the risk of bias was unclear in 1 out of 7 study. There was no high risk, or unclear risk of bias was observed in other items.

### Publication bias

**Fig 5:**
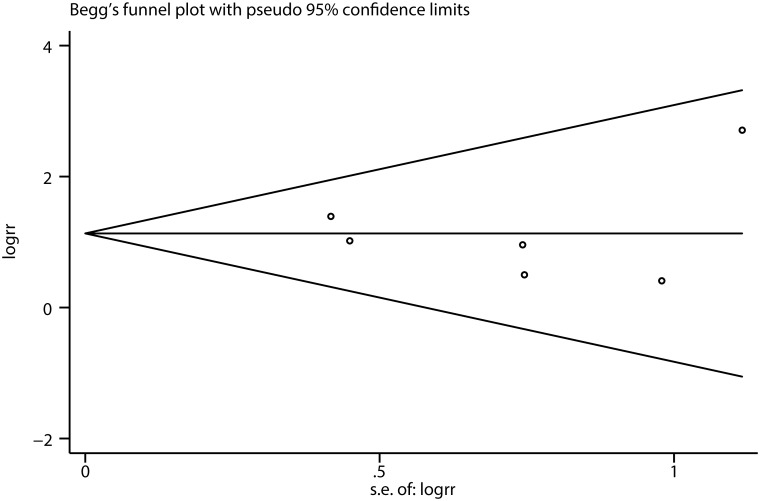
Begg funnel plot for publication bias test of each study.

The funnel plot was applied to evaluate the publication bias, indicating that there is no evidence for publication bias (Fig 5). In addition, this evidence is confirmed by a formal statistical test (***P*** = 0.967 of Egger’s test).

## Discussion

Based on the evidence of the current meta-analysis, sonothrombolysis for AIS might be debatable. When it comes to efficacy issues, we found that sonothrombolysis have a distinct benefit on the recanalization of occlusion, increasing the number of complete recanalization of occlusion, increasing complete or partial recanalization, and decreasing the frequency of the no recanalization than non-sonothrombolysis, which was in accordance with most other studies [5, 9, 14–17, 19, 25]. Although the specific mechanisms of sonothrombolysis were unclear [26], it has been hypothesized that TCD/TCCS could accelerate the movement of fluid around the thrombus so as to enhance the mixture of t-PA into the blood and consequently elevate the concentration of this drug near the occlusion [6, 7]. In addition, the pressure waves produced by TCD/TCCS may also augment the permeation of t-PA into the fibrin network and affect the binding of t-PA with fibrin directly [8]. Moreover, some studies believe that sustainable ultrasound exposure may result in vessel vasodilatation probably owing to the elevation of the activity of nitric oxide synthase, which leads to temporal vasodilation which improved reperfusion of local cerebral tissue [27, 28].

When it comes to safety issues, sonothrombolysis or non-sonothrombolysis might not influence the occurrence of ICH, disability or death at 3 months in patients with AIS. The results indicated that sonothrombolysis did not increase the adverse events compared with non-sonothrombolysis. In consideration of the sonothrombolysis hypothetical mechanisms, sonothrombolysis may improve the recanalization in short-term, but 3 months’ outcomes were impacted by more factors such as age, hyperlipidemia, and recurrence of stroke [6, 7, 27–30]. These factors may lead to the bias of the efficacy and safety results of 3 months’ outcomes, so that we may miss the long-term curative effect of sonothrombolysis.

The subgroup analysis on the basis of microbubbles demonstrated that sonothrombolysis without microbubbles was more effective and safer than non- sonothrombolysis thrombolysis through the subgroup data of excellent functional outcome (*P* = 0.049), good functional outcome (*P* = 0.032), NIHSS improvement ≥ 4 (*P* = 0.017) and death or disability (*P* = 0.011). However, in the sonothrombolysis with microbubbles subgroup, there were no significant differences between sonothrombolysis and non-sonothrombolysis in above mentioned outcome. In theory, the sonothrombolysis plus microbubble therapy should achieve better efficacy than non-sonothrombolysis. However, we got the opposite conclusion according to the sonothrombolysis plus microbubble subgroup anylsis. These results were different from many other articles and trials [9, 13, 19, 31, 32] It is possible that only two RCTs nvolve microbubbles, so that a small sample size is difficult to make a difference. In addition, microbubbles were supposed to work by absorbing energy, releasing energy, explosion [12, 33] and they might not only accelerate the dissolution of clots [34, 35] but also can cause the vascular endothelial damage directly [36]. Therefore, the value of sonothrombolysis plus microbubbles requires more randomized controlled trials to confirm.

Another subgroup analysis, compared with non-sonothrombolysis without t-PA subgroup, sonothrombolysis without t-PA subgroup showed more patients whose NIHSS decrease ≥ 4 (*P* = 0.030). However, when compared sonothrombolysis with t-PA subgroup with non-sonothrombolysis with t-PA subgroup, there is no significant difference in efficacy outcomes. Taken together, we concluded sonothrombolysis is a valuable method to treat AIS and t-PA also play an important role in thrombolysis. Therefore, t-PA is a favorable factor in sonothrombolysis for AIS.

From the rest of subgroup analysis, we found that sonothrombolysis might be more effective and safer for patients whose NIHSS scores at the beginning of the stroke were higher than 15 or the time of initial treatment was less than 150 min or ages were younger than 65 years, compared with non-sonothrombolysis group.

On the basis of our knowledge, most of previous systematic reviews and meta-analysis brought in several non-randomized types of research [19, 25, 34, 37, 38]. Combining all the results of no RCTs was heterogeneous so that these systematic reviews were flawed. Different from above-mentioned systematic reviews, all patients in the present meta-analysis were intervened by sonothrombolysis or non-sonothrombolysis and were randomized, which was the best way to divide risk factors equally over the two groups. This is the second meta-analysis about the sonothrombolysis all evidenced from RCTs (randomized clinical trials). In the first article Ricci et al. 2012 [5] included 5 RCTs, and our meta-analysis included 2 relatively novel RCTs, Dwedar et al. 2014 [16] and NOR-SASS 2017 [18], which was not used in preceding systematic reviews and meta-analysis. These two novel RCTs have a large sample size. Besides, we did further subgroup analysis including 5 relevant factors, which provide more comprehensive comparisons between sonothrombolysis and non-sonothrombolysis. With the data from new RCTs [16, 18], we might come to more definitive conclusions in order to guide clinical treatment. The following limitations of our meta-analysis should be noticed. Firstly, this meta-analysis was performed on base on the limited statistics. We only pooled 7 published RCTs^15, 19-24^ totally 549 patients were included (sonothrombolysis group, n = 302; control group, n = 247) to analysis the efficacy and safety of sonothromblolysis for AIS. Secondly, the included RCTs showed heterogeneity in the data of complete or partial recanalization (*I*^2^ = 42.2%), excellent functional outcome (*I*^2^ = 44.1%), good functional outcome (*I*^2^ = 60.6%) and death or disability (*I*^2^ = 34.7%). Although the sensitivity analysis demonstrated that all the consolidated statistics were stabilized, these disadvantages of the included studies could not be ignored. Thirdly, there was the possibility of selection bias in our meta-analysis, because we excluded some RCTs only with abstract reported in some meetings like Larrue et al. 2007 [22] and Dinia et al. 2016 [23]. Fourthly, our meta-analysis statistics could not represent all kinds of sonothrombolysis treatment, since we restricted it to TCD/TCCS-induced high-frequency ultrasound treatment (1.8–2 MHz).

In addition to the above limitations, sonothrombolysis itself has limitations in the treatment of AIS. Only patients with occlusion of the proximal middle cerebral artery can benefit significantly by sonothrombolysis. Therefore, the population suitable for treatment is limited. Furthermore, mechanical thrombectomy is currently recommended for the treatment of a large artery occlusion in patients with AIS. Sonothrombolysis is a relatively time consuming and operator-dependent procedure and presents many limitations, mainly technical, according to the presence of a temporal bone window to be performed. However, sonothrombolysis remains a treatment modality when patients admitted to stroke centers without endovascular competence until secondary transport to the intervention center.

## Conclusion

In conclusion, this meta-analysis demonstrated that sonothrombolysis treatment for AIS by TCD/TCCSc had significant efficacy on promoting recanalization and the upward tendency of the number of NIHSS improvement ≥ 4. However, it is not significantly different from other efficacy and safety outcomes. Some factors such as with t-PA, NIHSS > 15, treatment ≤ 150 min and Age ≤ 65 years may be potential advantages of sonothombolysis. These evidences can provide confident insights for further research on sonothombolysis in patients with AIS.

## Supporting information

S1 Fig The sensitivity analysis of good functional outcome. Fig 2E sensitivity analysis was performed to detect the source of statistical heterogeneity. which showed that all of the consolidated results were stable.
(TIF)

S1 File PRISMA 2009 checklist. (DOC)
