# Supplementary figures and images for: Efficacy and safety of sonothombolysis versus non-sonothombolysis in patients with acute ischemic stroke: A meta-analysis of randomized controlled trials
Source: PLoS One. 2019 Jan 9;14(1):e0210516. doi: 10.1371/journal.pone.0210516 (PMC6326494; doi:10.1371/journal.pone.0210516)

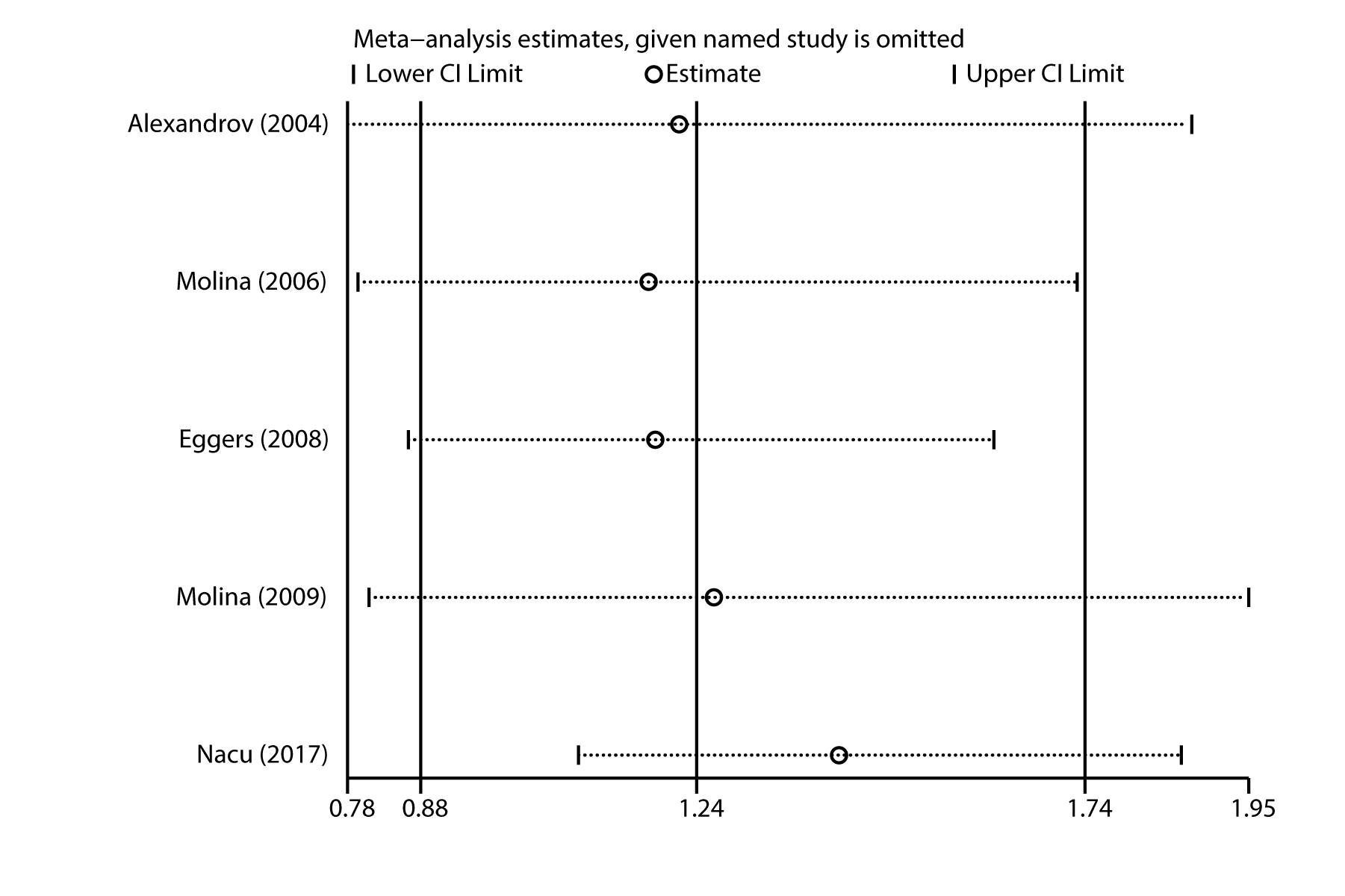

Supplement: S1 Fig — Fig 2E sensitivity analysis was performed to detect the source of statistical heterogeneity. which showed that all of the consolidated results were stable. (TIF) [file pone.0210516.s001.tif]
